# Cancer Chemotherapy Related Cognitive Impairment and the Impact of the Alzheimer’s Disease Risk Factor *APOE*

**DOI:** 10.3390/cancers12123842

**Published:** 2020-12-19

**Authors:** Harvey R. Fernandez, Ashima Varma, Sarah A. Flowers, George William Rebeck

**Affiliations:** Department of Neuroscience, Georgetown University, Washington, DC 20057, USA; Harvey.Fernandez@georgetown.edu (H.R.F.); av795@georgetown.edu (A.V.); Sarah.Flowers@georgetown.edu (S.A.F.)

**Keywords:** Apolipoprotein E, chemotherapy, cognitive impairment, *APOE*, Alzheimer’s disease, inflammation, blood brain barrier, neurogenesis

## Abstract

**Simple Summary:**

Research into the causes and potential treatments for cancer-related cognitive impairment has increased greatly over the past several years. Advances have been made in studies related to human neuropsychology and animal models of behavior. Findings from both types of studies implicate a role of the genetic risk factor *APOE4* in cancer-related cognitive impairment. *APOE4* is the strongest genetic risk factor for Alzheimer’s disease, and this convergence across disparate research approaches now provides a great opportunity for insight into mechanisms of both conditions. This review provides an overview of potential mechanisms that could account for aspects of cancer-related cognitive impairment, and how they could be affected by the *APOE* genotype.

**Abstract:**

Cancer related cognitive impairment (CRCI) is a serious impairment to maintaining quality of life in cancer survivors. Cancer chemotherapy contributes to this condition through several potential mechanisms, including damage to the blood brain barrier, increases in oxidative stress and inflammation in the brain, and impaired neurogenesis, each of which lead to neuronal dysfunction. A genetic predisposition to CRCI is the E4 allele of the Apolipoprotein E gene (*APOE*), which is also the strongest genetic risk factor for Alzheimer’s disease. In normal brains, APOE performs essential lipid transport functions. The APOE4 isoform has been linked to altered lipid binding, increased oxidative stress and inflammation, reduced turnover of neural progenitor cells, and impairment of the blood brain barrier. As chemotherapy also affects these processes, the influence of *APOE4* on CRCI takes on great significance. This review outlines the main areas where *APOE* genotype could play a role in CRCI. Potential therapeutics based on APOE biology could mitigate these detrimental cognitive effects for those receiving chemotherapy, emphasizing that the *APOE* genotype could help in developing personalized cancer treatment regimens.

## 1. Introduction—Cancer Related Cognitive Impairment after Chemotherapy

While the increasing success in treating cancer improves survivorship, the detrimental effects of cancer chemotherapy on the central nervous system (CNS), including neurotoxicity and reduced cognitive ability, have been observed for decades [1,2,3,4,5]. Cancer related cognitive impairments (CRCI) affect memory, verbal ability, and executive functions [6,7]. Mitigating the CNS side effects would substantially improve prognoses by increasing quality of life [8,9,10,11]. While CRCI is a broad field that includes cognitive problems suffered by cancer patients regardless of treatment [12], this review will focus on the cognitive impairments induced after chemotherapy.

Gross CNS impacts after cancer treatments are observed in MRI scans [13]. Decreased gray matter density was found in patients one month after treatment, some of which persisted even one year later [14] and these changes to brain structures correlated with cognitive difficulties [15]. CRCI occurs in survivors of many cancers [16], although breast cancer survivors have been the most extensively characterized. Neuropsychological analyses of breast cancer survivors found that chemotherapy treatment was associated with significant reductions in verbal memory and psychomotor function persisting for up to 10 years [17]. Breast cancer survivors administered chemotherapy had significantly lower scores in visual memory and visuospatial function than survivors given local therapy and healthy controls [18]. Five to ten years post-treatment, breast cancer survivors who had completed systemic treatment with chemotherapy performed significantly worse on short term memory tasks, which correlated with reductions in the resting metabolism levels of several brain regions as measured by [F-18] fluorodeoxyglucose (FDG) positron emission tomography (PET) scans [19]. Cytostatic chemotherapies were also associated with structural changes in the white matter in regions of the brain involved in cognitive processes [20], which correlate with worse performance in verbal memory tests [20]. Over the long term, chemotherapy is associated with white matter deficiencies and reduced scores on cognitive tests, as well as gray matter alterations over nine years [21] and 21 years [22]. Overall, the cognitive impairments induced by chemotherapy are persistent and involve changes in brain structure and physiology.

### Involvement of APOE in CRCI

It is unknown what factors predispose a person to CRCI, although several genetic studies have identified the *APOE* gene [5,23,24,25]. *APOE* is the strongest genetic risk factor for Alzheimer’s disease (AD) [26,27,28]. *APOE* encodes the apolipoprotein E protein, a 299 amino acid, secreted *O*-glycoprotein that transports lipids in the periphery and CNS [29]. In the CNS, APOE affects processes such as neuronal health, inflammation, oxidative stress, and clearance of the AD pathogenic protein Aβ [30,31,32]. APOE also plays important roles in the neurovascular system and the blood brain barrier [33], a structure crucial to preventing many chemotherapeutics direct access to the CNS. There are three *APOE* alleles that produce isoforms with single amino acid differences: *APOE2* (Cys112, Cys158), *APOE3* (Cys112, Arg158), and *APOE4* (Arg112, Arg158). Compared to the most common *APOE3* allele, *APOE2* reduces risk of AD by up to 50% and *APOE4* increases AD risk three-fold in heterozygotes and 14-fold in homozygotes [34]. *APOE4* is common, with approximately a quarter of the US population carrying an *APOE4* allele [35]. *APOE4* allele frequencies differ across ethnicities around the world [36]: 14% in Caucasian Americans [35], up to 40% in African Americans [37], 10% in Japan and Italy [38,39], and up to 20% in Sweden [40].

Several studies have addressed the influence of the *APOE* genotype on risk of CRCI (described in Table 1). For breast cancer survivors treated with chemotherapy five years post diagnosis, *APOE4* carriers had worse visual memory and spatial ability [23]. *APOE4* breast cancer patients with no smoking history had significantly lower scores on measures of processing speed and working memory at 1, 6, and 18 months post-chemotherapy [24]. *APOE4* carriers who received chemotherapy for testicular cancer had a significantly lower global cognitive score based on a battery of cognitive tests [25]. Finally, *APOE4*-positive breast cancer survivors assigned cytostatics had lower scores on tests measuring attention, processing speed and executive function, as well as learning and memory compared to controls or those given only hormone therapy, one to two years post-treatments [5]. Together, these studies show that the *APOE* genotype has an impact on cognitive decline following chemotherapy.

In addition to these clinical studies, preclinical models from our lab using transgenic knock-in mice expressing the human *APOE* alleles support a role for *APOE* in CRCI. Such mouse models allow for a rigorous comparison of *APOE3* and *APOE4*, controlling for variables such as diet, age, and chemotherapy regimen. Four to six-month old female *APOE4* mice treated with the common breast cancer chemotherapeutic doxorubicin showed induced deficits in spatial learning and reduced grey matter volumes compared to untreated *APOE4* mice, as well as treated and untreated *APOE3* mice [41]. At 12 months of age, treated *APOE4* mice again showed impairments in spatial memory compared to untreated *APOE4* mice. *APOE3* mice showed no deficits post-treatment [42]. These studies support the human genetic studies demonstrating that the *APOE* genotype plays a role in the detrimental CNS side effects of chemotherapy (Table 1).

## 2. Molecular Mechanisms of CNS Damages in CRCI

The numerous neurophysiological processes affected by APOE suggest many pathways for its influence on the mechanisms related to CRCI. Several possible CNS mechanisms for CRCI influenced by the *APOE* genotype are reviewed here, including blood brain barrier compromise, oxidative stress, inflammation, and deleterious effects on adult neurogenesis.

### 2.1. Blood Brain Barrier

The blood brain barrier (BBB) relies on tight junctions between endothelial cells, membrane-bound efflux proteins, the glia limitans, and a relative lack of endothelial fenestrations in the CNS [43]. As most chemotherapeutics do not readily cross the BBB, the cognitive effects of CRCI are not usually associated with direct CNS damage from the drugs. Rather, the effects of chemotherapy that induce peripheral damage can affect the integrity of the BBB, which could then result in CNS damage as peripheral molecules enter.

Oxidative stress from reactive oxygen species (ROS) induce membrane protein and lipid oxidation (discussed in detail below), which causes dysfunction of the BBB. ROS can impact expression of tight junction proteins [44], affect cytoskeletal structure, and activate matrix metalloproteinases (MMPs), all affecting BBB permeability [45]. The impact of ROS on in vitro models of the BBB included activation of MMPs, which reduced structural integrity, increased permeability, and allowed leukocytes to migrate through [46,47]. One cancer chemotherapeutic agent, oxaliplatin, directly affected expression of the tight junction proteins zonula occludens-1 (ZO-1) and F-actin in an in vitro model [48]. In vivo, the induction of enzymes that increase ROS production (NAPDH oxidase (NOX1) and inducible nitric oxide synthase (iNOS)) correlated with a reduction in expression of tight junction proteins [49]. Inflammatory cytokines also impact the integrity of the BBB. Tumor necrosis factor alpha (TNFα) is induced by many chemotherapeutics (also discussed below) and alters BBB integrity by upregulating MMPs [50]. Cytokines also reduce BBB integrity through effects on tight junctions and multiple components of the neuroimmune system [51].

#### *APOE* Genotype and the Blood Brain Barrier

The increased susceptibility of *APOE4* carriers to CRCI may be due in part to APOE’s involvement in the health of the BBB [33]. A direct role for *APOE* in BBB function was initially demonstrated in *APOE* knockout mice, which showed an infiltration of plasma antibodies throughout the CNS [52]. In the CNS, astrocytes and microglia are the main producers of APOE, but vascular cells (smooth muscle and endothelial cells) and pericytes (cells associated with small vessels) also express APOE [33].

Mouse knock-in models of the human *APOE* alleles are useful for comparisons among human *APOE* alleles as well as for comparisons with wildtype mice. These models show levels and patterns of *APOE* expression similar to wildtype mice and humans [53,54]. Wildtype mice show more similarity in cerebral blood flow and neurovascular density to *APOE3* than *APOE4* mice [55], consistent with the idea that *APOE3* mice reflect normal brain conditions. *APOE4* mouse brains showed less collagen IV, an important basement membrane protein, and an increased penetration of plasma proteins [56]. *APOE2* and *APOE3* mouse brains were impermeable to fluorescently labeled dextran and multiple plasma proteins, while both *APOE4* and *APOE* KO allowed leakage into the brain [57]. One mechanism identified involved the pro-inflammatory cytokine CypA, which had previously been shown to cause vascular damage in *APOE* KO mice [57]. Both *APOE4* and *APOE* KO mice had five- to six-fold elevated levels of CypA in cerebral micro vessels, which was responsible for increased BBB permeability through the activation of MMP9. BBB breakdown correlated with a reduction in tight junction proteins that are MMP9 substrates. The proper regulation of CypA levels required the binding of astrocyte-derived APOE3 to low density lipoprotein receptor 1 (LRP1), of which APOE4 binding was deficient [57]. In in vitro assays, APOE4 had significantly less effect at attenuating MMP activity than APOE2 or APOE3 [58]. Postmortem analysis of human brain AD tissue showed accelerated pericyte and microvascular breakdown in *APOE4* carriers, and increased presence of serum proteins in the CNS, indicative of BBB breakdown [59]. There was also a corresponding increase in CypA and MMP9 levels [57]. Finally, an *APOE* AD mouse model showed that *APOE4* effects on BBB leakage were exacerbated by the presence of the Aβ peptide that accumulates in brains of AD patients [60].

In vitro models provide more mechanistic studies of the BBB. One model of brain endothelial cells and pericytes from wildtype mice, combined with astrocytes harvested from *APOE3* and *APOE4* knock-in transgenic mice, showed that APOE4 impaired BBB integrity and altered phosphorylation of the tight junction protein occludin [61]. The tight junction integrity was dependent on LRP1 binding, as treatment of the *APOE3* model with an LRP1 inhibitor caused leakage of the BBB to levels similar to that of the *APOE4* containing model [61]. Pericyte APOE4 reduced induction of endothelial cell extracellular matrix production and BBB formation [56].

Breakdown of the BBB correlates with cognitive decline with aging and AD in humans [62,63,64]. The *APOE4* genotype was associated with a thinning of the microvascular basement membrane in AD patients [65]. The plasma protein prothrombin was present within micro vessels in the brain of AD patients, and this leakage through the BBB was more common in individuals with at least one *APOE4* allele [66]. An examination of regional cerebral blood flow in healthy non-demented subjects over a period of eight years showed that the declines over this time period were more pronounced in *APOE4* carriers [67]. High resolution MRI scans illustrated that *APOE4* carriers had elevated BBB breakdown that was evident even in cognitively normal patients, and breakdown was further exacerbated in cognitively impaired subjects [68]. Higher levels of soluble platelet derived growth factor receptor-β (sPDGFRβ) was found in CSF of *APOE4* but not *APOE3* carriers [68]; PDGFRβ induces BBB restoration [69] and this shed form of PDGFRβ inhibits normal PDGFRβ signaling [70]. sPDGFRβ levels predicted cognitive decline, as well as increased BBB permeability and levels of plasma protein leakage into the CSF [68].

**Figure 1:**
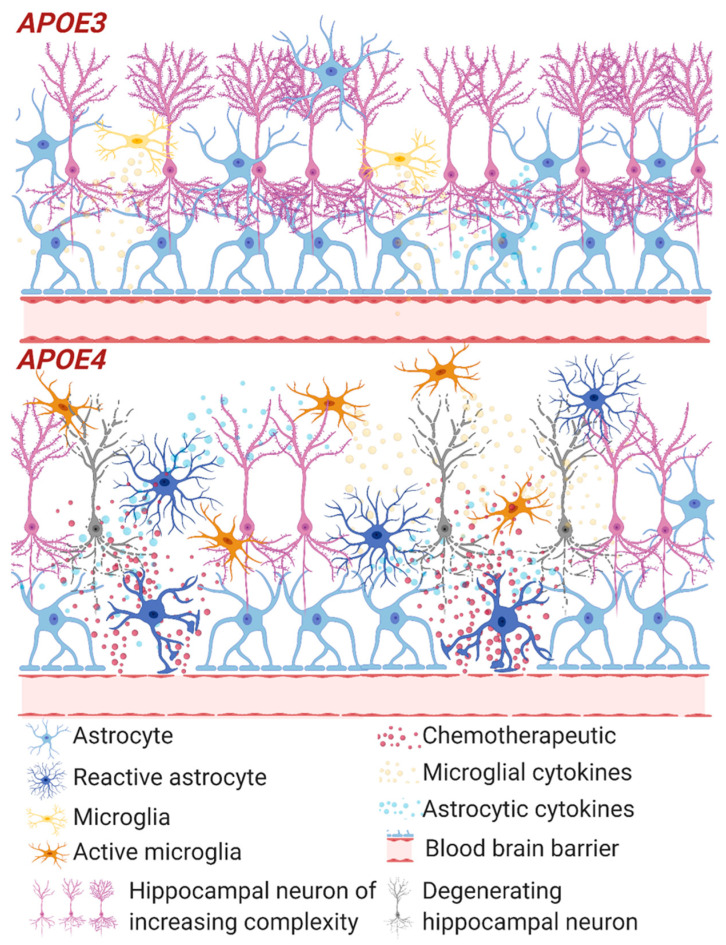
Inflammation and the blood brain barrier with chemotherapy. Summary of impacts of chemotherapeutics on *APOE3* and *APOE4* neural tissue and the blood brain barrier (BBB). *APOE4* show thinning basement membrane and impaired tight junctions leading to increased BBB permeability and release of chemotherapeutic (red particles) into the brain compartment, not apparent in *APOE3*. This impairment leads to increasing the already heightened immune and oxidative stress response in *APOE4*, increasing reactive astrocytes and active microglia, further increasing BBB breakdown. Ultimately this results in an environment that increases neurodegeneration (grey neurons). Although chemotherapeutic agents are unable to cross the secure *APOE3* BBB, they are still able to induce a cytokine response close to the BBB which may damage the integrity of the tight junctions.

Thus, important components of the impact of *APOE4* on BBB function and integrity have been identified and these mechanisms could underlie impairment of brain function by chemotherapy (Figure 1). BBB disruption related to *APOE4* and chemotherapy could allow greater penetration of neurotoxic drugs or exacerbate the deleterious effects of processes such as oxidative stress and inflammation.

### 2.2. Oxidative Stress

Most of the cytostatic agents used to treat cancer induce oxidative stress, whether indirectly or directly, contributing to the neurotoxicity of many of these agents [71]. Cyclophosphamide (CPA) [72], doxorubicin (DOX) [73], and methotrexate (MTX) [74] generate oxidative stress in the CNS through imbalanced ROS levels, demonstrated by various markers. CPA increased cortical levels of malondialdehyde, conjugated dienes, and hydroperoxides [72,75]. In a breast cancer rodent model, CPA and DOX caused increased expression of oxidative stress related genes such as glutathione peroxidase 1 (GPX1), peroxiredoxin 1 (PRDX1), and NF-ΚB in the hippocampus [76]. An anti-oxidant compound mitigated the cognitive effects of chemotherapies [77], indicating that oxidative stress is one mechanism for CRCI. Co-administration of metformin as an antioxidant with CPA protected mice from the memory deficits associated with hippocampal damage [78]. The anti-stroke drug edavarone, which has anti-oxidant activity, blocked the reductions in superoxide dismutase, catalase and glutathione activities, and ameliorated the memory and motor coordination impairments induced by CPA [79]. A mimetic of superoxide dismutase used to reduce oxidative stress corrected cognitive behavioral deficiencies and neuronal cell changes induced by CPA, DOX, and paclitaxel in mice [80]. MTX-induced oxidative stress was relieved by co-administration of melatonin, an anti-oxidant, which resulted in restoration of spatial cognitive function in rats [81]. Finally, DOX increased protein oxidation and lipid peroxidation in the brains of mice [73], and the anti-oxidant activity of insulin reduced oxidative stress and improved cognitive function [82].

Several mechanisms could account for chemotherapy-induced oxidation. DOX increased Ca^2+^-mediated induction of the permeability transition pore (PTP) in brain mitochondria [83]. A PTP increase in mitochondrial membrane permeability can lead to mitochondria swelling and rupture, thereby inducing oxidative stress in the brain [83]. MTX, a folate analog, induces oxidative stress via interactions with enzymes in the folate metabolic pathway, causing elevated levels of malondialdehyde in the cerebellum in rats [84]. DOX increased TNFα production, inhibiting murine brain mitochondrial respiration and increased oxidative stress [85]. Similarly, rats injected with DOX showed increased carbonylation of brain proteins, likely mediated by increases in TNFα [86]. The involvement of TNFα in DOX-induced oxidative stress was confirmed through the use of TNFα knockout mice. In these animals the oxidative stress was alleviated, and mitochondrial activity was preserved [87]. Pro-inflammatory molecules such as TNFα thus allow certain chemotherapeutic agents to exert oxidative damage throughout the CNS, despite some of these agents being unable to cross the BBB.

In addition to animal studies, studies in human tissue show evidence of chemotherapy-related oxidative stress. Significant increases in TNFα and protein carbonyls following DOX treatment were observed in the plasma of patients [88]. CSF of acute lymphoblastic leukemia (ALL) patients showed evidence of lipid peroxidation, where the dose of MTX correlated with the levels of oxidized phosphatidylinositol [89]. While MTX allows good survival from childhood ALL, up to 40% of survivors show cognitive deterioration [89]. Polymorphisms in oxidative stress/neuroinflammation-related genes (NOS3, COMT, SLCO2A) were associated with poor neurocognitive performance on a series of behavioral tests after MTX [90]. This genomic analysis, along with the other studies mentioned, indicate that oxidative damage can contribute to the CRCI.

#### *APOE* Genotype and Oxidative Stress

*APOE* genotype affects susceptibility to oxidative stress, potentially exacerbating CRCI. *APOE4* carriers had higher levels of certain ROS in plasma than control subjects [91]. In a postmortem study of AD patients, *APOE4* brains showed greater levels of oxidative stress, including elevated lipid peroxidation in the hippocampus, and lower levels of catalase activity, glutathione peroxidase activity, and glutathione throughout the cortex [92]. Levels of thiobarbituric acid-reactive substances (TBARS), a measure of lipid peroxidation, were highest in *APOE4* and lowest in *APOE2* brains [93]. *APOE4* carriers also had higher hydroxyl content in the blood, indicating a systemic increase in oxidative stress [94].

In a mouse model of traumatic brain injury (TBI) which induces oxidative stress, there was greater expression of anti-oxidant related genes in *APOE3* and higher levels of oxidative stress markers by immunohistochemitry in *APOE4* mice [95]. The *APOE* knock-in mice are also useful for elucidating synaptic structure and function through use of synaptosomes. Synaptosomes isolated from the *APOE4* mice had the highest levels of ROS, protein and lipid oxidation, followed by *APOE3* and then *APOE2* [96]. The proteomes of synaptosomes from *APOE4* mice had elevated oxidative stress markers compared to *APOE3* mice [97].

In vitro studies showed that the three APOE isoforms provided different levels of protection against oxidative stress and toxicity caused by Aβ peptides, with APOE4 providing the least protection and APOE2 the most [98]. Several mechanisms have been proposed for the relationship between *APOE4* and oxidative damage. *APOE* KO neurons had significantly increased levels of lactate dehydrogenase, decreased levels of SOD activity and increased intracellular Ca^2+^ levels when treated with APOE4 protein, compared to APOE3 [99]. APOE4 induced increased susceptibility to Ca^2+^-mediated neuronal apoptosis by increasing CaMKII phosphorylation and activation of caspase 3 [99]. The lack of cysteine residues (and their replacement with arginine) in the APOE4 molecule may contribute to induction of oxidative damage. Specifically, the neurotoxic 4-hydroxynonenal (HNE) molecule (which is produced as a result of lipid oxidation) can covalently bind to cysteine residues on APOE2 and APOE3 and prevent further damage to neighboring neurons [100]. APOE4 cannot bind HNE, allowing it to cause oxidative damage to neuronal proteins and eventually cell death [100].

Together, clinical and experimental findings show that common chemotherapeutic agents induce oxidative stress, as measured in a number of biological processes. *APOE4* has broad impacts on oxidative stress in the brain, potentially amplifying damage introduced by chemotherapeutic treatments. These CNS damages could be mitigated by antioxidant treatments, protecting against neurotoxicity and the development of CRCI.

### 2.3. Inflammation

Inflammation resulting from cytostatics could be involved in promoting or exacerbating CRCI. Neuroinflammation is a critical component of neurodegeneration [101], and pro-inflammatory cytokines such as TNFα and interleukins are associated with impairments in memory and cognition. Chemotherapeutics that do not cross the BBB induce pro-inflammatory cytokines in the periphery, which could cross the BBB (by diffusion through a damaged barrier or by receptor mediated endocytosis) and elicit gliosis associated with CNS inflammation [102].

#### 2.3.1. Inflammatory Cytokines

Cytostatics can have differing effects on the many markers of inflammation [103]. Patients receiving DOX for early stage breast cancer showed that the blood levels of cytokines IL-6, IL-8 and monocyte chemoattractant protein 1 (MCP-1) increased. These same cytokines decreased for CPA, MTX and fluorouracil. Breast cancer survivors who received surgery plus chemotherapy had significantly elevated levels of TNFα activity compared to those who had only surgery [104]. The cytokines IL-6 and TNFα were also both elevated (an average of 5 years post-surgery and chemotherapy) and associated with decreased verbal memory performance and lower hippocampal volume [105]. TNFα decreased over the first year, which correlated with a reduction in self-reported memory complaints. Brain metabolism (as measured by FDG PET) negatively correlated with TNFα in specific brain regions [104]. Cognitive response speed was negatively associated with increases in IL-1β plasma levels, but positively correlated with IL-4 (indicating a protective effect of IL-4) [106]. Higher concentrations of IL-1β and IL-6 accompanied lower self-perceived cognitive performance, while patients with higher IL-4 reported less severe cognitive disturbance. The levels of sTNF-RII (a marker of TNFα activity) and MCP-1 showed a significant association between elevated levels of sTNF-RII and reduced short term memory [106].

For B-cell non-Hodgkin lymphoma, the first line drugs rituximab and bendamustine (RB) increased serum IL-6 more than rituximab and CPA, DOX, vincristine, and prednisone (R-CHOP), and the levels of IL-6 correlated with increased fatigue three months after the end of chemotherapy [107]. Patients receiving RB had significantly lower scores in cognitive tests than a healthy cohort, while the R-CHOP trended lower than the controls [107], another example of one chemotherapy type producing varying responses. An examination of leukoencephalopathy in ALL survivors after treatment found that MTX exposure correlated with persistent increases in neuroinflammation (including astrogliosis and microglial activation) and increased neuronal damages resulting from this neuroinflammation were proposed to cause the associated cognitive problems [108]. This work across survivors of breast cancer, B-cell non-Hodgkin lymphoma, and ALL shows that chemotherapy produces strong (and varied) inflammatory responses impacting memory, cognition, and even brain structure.

Animal models allow more direct identification of mechanisms related to CRCI. Although DOX does not cross the BBB, it results in increases in peripheral and brain TNFa [85]. The elevation in brain TNFα levels was associated with reduced mitochondrial activity, and a causal connection was demonstrated by the use of anti-TNFα antibodies alleviating these effects [85]. DOX, docetaxel, and CPA treatment resulted in elevation of IL-6 and TNFα in serum and the hippocampus, as well as reductions in the anti-inflammatory cytokines IL-4 and IL-10 [109]. IL-6 and TNFα levels showed strong inverse correlations with mouse performance in water maze cognitive tests, combined with reduced signal in manganese enhance magnetic resonance imaging measuring hippocampal neuronal activity [109]. The levels of these pro-inflammatory cytokines also showed robust correlation with dendritic spine loss, a marker of reduced neuronal complexity [109]. DOX also increased TNFα levels in the brains of rats, with the robust inflammatory response further evidenced by significant increases in the levels of prostaglandin E2 and cyclooxygenase 2 (COX-2) [110].

Agents countering inflammation have been used in rodent models to alleviate behavioral effects of chemotherapy. Combined breast cancer chemotherapies of CPA, MTX, and 5-fluorouracil resulted in increased levels of TNFα, IL-1β, and COX-2 in rat brain, while levels of the anti-inflammatory cytokine IL-10 decreased [111]. Problems with inflammation, myelin levels, and cognitive scores were all alleviated by the anti-inflammatory COX-2 inhibitor NS-398 [111]. DOX treatment resulted in inflammatory responses including the activation of the NF-ΚB pathway as well as reduced spatial learning and memory [112]. DOX led to elevated TNFα, PGE-2, and COX-2 levels in the brain, which was counteracted by polydatin (PLD), an anti-inflammatory and anti-oxidant [112]. Chemotherapy induced neuronal apoptosis in the hippocampus, along with the reduced learning and memory, were alleviated by PLD which also normalized the levels of NF-ΚB pathway intermediates. Despite a different mechanism of action, paclitaxel (which works by destabilizing microtubules as opposed to damaging DNA) also impairs cognition [113]. Paclitaxel induced inflammation in the hippocampus, with increases in TNFα and IL-1β [114]. The TNFα inhibitor thalidomide reduced inflammation and neuronal apoptosis and memory and spatial learning deficits [114].

These studies show that chemotherapy effects on brain function are related to inflammation and suggest interesting directions for treatments aimed at reducing CRCI.

#### 2.3.2. Gliosis

The primary regulators of neuroinflammation in the brain are two glial cell types, astrocytes and microglia [115]. Many brain insults induce activation and proliferation of glial cells as part of the pathways to repair damages and clear cellular debris. Importantly, glia are the main source of brain APOE.

In mouse models, DOX treatment induced widespread astrogliosis, which correlated with oxidative stress, increased IL-6 and TNFα, while reducing IL-10 and cognitive impairments [116]. Treatment of mice with the colony stimulating factor receptor 1 (CSF1R) inhibitor PLX5622, which depleted over 95% of brain microglia, caused reductions in multiple pro-inflammatory cytokines and behavioral impairments caused by DOX [117]. A role for the third type of glial cell, oligodendrocytes, was indicated by MTX treatment associated with problems with myelination and CRCI [118]. Analysis of human postmortem brain samples showed oligodendrocyte lineage cells were depleted in subjects that had undergone chemotherapy. A mouse model was developed to mimic the regimens in these patients, and it demonstrated deficits in oligodendrocyte precursor cell (OPC) numbers even 6 months after treatment cessation [118]. Reduced myelin sheath thickness corresponded to behavioral deficits similar to those that affect humans (attention, short term memory). MTX also caused activated microglia in the brain white matter, which also persisted 6 months later. Brain injury—as defined by activated astrocytes—lowered oligodendrocyte precursors, and reduced myelin sheath thickness were alleviated by depletion of the inflammatory microglia [118]. These various reports demonstrate the crucial role of glia in promoting adverse effects of chemotherapy on brain functions.

#### 2.3.3. *APOE* Genotype and Neuroinflammation

Many AD genetic risk factors are directly related to processes important in neuroinflammation, including *TREM2, CD33, CR1*, and *APOE* [119]. In addition to its role in cholesterol homeostasis, *APOE* has profound effects on neuroinflammation, with the APOE4 protein promoting a pro-inflammatory environment, whether under normal conditions or in response to injury and disease [115]. Analyses of post-mortem AD patient brains found that *APOE4* subjects had significantly increased activated microglia [120] and astrogliosis [121]. The *APOE2* genotype was associated with a pattern of microglial protein expression that correlated with good cognition, while the *APOE4* genotype was associated with proteins correlating with poor cognition [122]. Increased inflammatory cytokines were present in the plasma of even healthy *APOE4* carriers [123], and elevated cytokines combined with the *APOE4* genotype to increase risk of AD [124]. Greater cytokine production in *APOE4* carriers was observed from the ex vivo stimulation of blood from patients with toll-like receptor ligands, as well as in those who had challenges with intravenous lipopolysaccharide (LPS) [125]. CCL23, a pro-inflammatory chemokine associated with cerebral damage [126], was increased in the plasma of *APOE4* carriers in patients with mild cognitive impairment (MCI) and AD [127]. The CSF of *APOE4* MCI and mild AD patients also had significantly increased amounts of IL-4, IL-6, and IL-8 and granulocyte colony stimulating factor [128]. A proteomic analysis of postmortem cortical samples revealed that inflammation was the biological pathway most impacted by *APOE* genotype in AD patients [129].

The importance of the *APOE4* allele was vividly illustrated by the use of CRISPR editing technology in human induced pluripotent stem cells (iPSCs). Microglia derived from genetically identical *APOE3* and *APOE4* iPSCs [130] showed *APOE4* microglia had elevated expression of the transcription factor IRF8 regulating multiple genes required for microglial activation [130].

The transgenic *APOE* knock-in mice have also provided much data on the role of *APOE4* in inflammation. *APOE4* mice have stronger inflammatory gene expression responses to LPS injected into the brain [131]. An increased inflammatory response to LPS injection in *APOE4* mice also led to stronger gliosis and more synaptic protein loss [132]. A broad hippocampal microgliosis and astrogliosis in *APOE4* mice compared in *APOE3* mice was associated with accumulation of the neurotoxic intracellular Aβ [133]. LPS treatment of primary *APOE4* microglial cultures led to more extensive neuron damage compared to *APOE3* microglia [134], with similar experiments entailing in vitro exposure to LPS inducing significantly elevated pro-inflammatory cytokine production in *APOE4* microglia [135]. The induction of acute brain injury from needle penetration resulted in greater inflammatory responses as measured by reactive astrocytes and microglia in *APOE4* mice [136]. Control *APOE4* mice had a lower dendritic spine density [137,138], likely a result of increased inflammation, as this phenotype was alleviated with nonsteroidal anti-inflammatory drug treatment [139].

The combination of AD-related transgenes along with *APOE* knock-in further demonstrated the increased inflammation in *APOE4* mice. Transgenic mice containing five familial AD mutations along with *APOE4* had higher cortical cytokine and microglial reactivity [140], while *APOE4* mice with the P301S tau transgene had significantly higher levels of TNFα and reduced neuronal viability compared to *APOE2* or *APOE3* [141]. The impacts of chemotherapy on neuroinflammation, combined with the profound *APOE4*-dependent increase in the susceptibility to inflammation as a result of brain injuries, provide a robust link between the *APOE* genotype and CRCI.

### 2.4. Impaired Neurogenesis

Current cancer treatments are aimed at reducing atypical cell division associated with malignancy. However, they could lead to unintended impairment of neural stem cell proliferation, particularly if chemotherapeutic agents cross the blood brain barrier at times of damage by oxidative stress or inflammation.

The process of neurogenesis involves neural progenitor cells developing and maturing into new neurons. The adult brain retains two regions with prominent neurogenesis: the sub-granular zone in the dentate gyrus of the hippocampus and the subventricular zone. Cytostatics decrease neurogenesis and progenitor proliferation in these areas [142]. Cisplatin, carmustine, and AraC each decreased cell division in the dentate gyrus and the subventricular zone of mouse brains, and this pattern persisted for weeks even after treatment ceased [143]. CPA decreased the number of dentate gyrus cells expressing Ki-67 and doublecortin (DCX), markers of neurogenic cells [144]. CPA also reduced DCX levels in rats, decreased numbers of newborn granule cells, and caused less dendritic branching and shorter dendrite length, all of which correlated with reduced memory and learning capability [145]. A chronic regimen of CPA at a lower dose in rats, in contrast, did not affect neurogenesis [146]. Both CPA and DOX treatment of rats significantly reduced mature (DCX) or immature (Ki-67/NeuN) neurogenesis markers [147]. Interestingly, while both drugs affected neurogenesis, only CPA resulted in increased microglial activation [147]. The combination of DOX, CPA and 5-fluorouracil (5-FU) reduced neurogenesis and caused memory impairments in mice [148]. This same drug combination resulted in similarly reduced neurogenesis in rats and also caused changes in chromatin remodeling in the hippocampus, consistent with the observed memory and spatial learning deficits [149].

Other cytostatics, such as MTX or 5-FU, also reduced neurogenesis. MTX inhibited neurogenesis [150,151] and caused decreased white matter density in the lateral corpus callosum and memory impairments [152]. The combination of MTX and 5-FU led to significant hippocampal and frontal lobe volume changes alongside impaired neurogenesis and cognitive impairment [153]. 5-FU alone lowered levels of brain-derived neurotrophic factor and reduced spatial working memory [150]. Temozolomide, a cytostatic agent used for CNS tumors, also reduced adult hippocampal neurogenesis [154]. Unlike many of the drugs mentioned, temozolomide directly accesses the brain.

Together, these studies establish that chemotherapeutic agents can decrease neural progenitor development in critical proliferative locations. Damage to cells in these germinal areas is a key mechanism by which chemotherapy could exert long-lasting, neurotoxic effects, which in turn could impair processes such as brain plasticity and cognition [143,148,153].

#### *APOE* Genotype and Hippocampal Neurogenesis

Adult neurogenesis is important for the proper functioning of the hippocampus, a region affected early in the pathogenesis of AD [155,156]. Neural progenitor cells in the dentate gyrus require *APOE* expression for proper self-renewal capability. *APOE* knock-out resulted in loss of neural progenitor cells [157], as well as an increase in the proliferation of astrocytes [158]. In *APOE4* mice there was increased proliferation of neural stem cells, but decreased neuronal maturation and neurogenesis compared to *APOE3* mice, demonstrating that proper differentiation was also dependent on *APOE* [158]. Furthermore, dendritic development of newborn hippocampal neurons was impaired by *APOE4*—the dendritic length and branch number were significantly lower in *APOE4* mice compared to *APOE3. APOE4* knock-in mice displayed a decrease in hippocampal interneurons, resulting in learning and memory deficits [159]. Dysfunction of these neurons leads to impairment of adult hippocampal neurogenesis in mice [158]. In both transgenic *APOE3* and wildtype mice, neurogenesis in the dentate gyrus is induced by environmental stimulation. In contrast, the response in *APOE4* mice was the reduction of cell proliferation [160].

Analysis of the effect of the *APOE* genotype during aging showed that neurogenesis in young (10–12 weeks) *APOE4* male and female mice was lower than in *APOE2* and *APOE3* mice [161] and effects in aged mice were limited to females [161]. In young mice, TBI induced neuronal proliferation in the hippocampus, but caused an attenuated response in *APOE4* mice, while *APOE* KO mice displayed a complete lack of proliferation [162]. *APOE* KO and *APOE4* mice also showed impaired dendritic arborization and diminished spine density in newborn granule neurons after TBI compared to *APOE3* [163]. These studies show that impairment of neurogenesis in *APOE4* carriers continues throughout the lifetime and after damage, particularly in females. The particular sensitivity of females may be especially significant considering the susceptibility of breast cancer survivors to CRCI [164].

**Figure 2:**
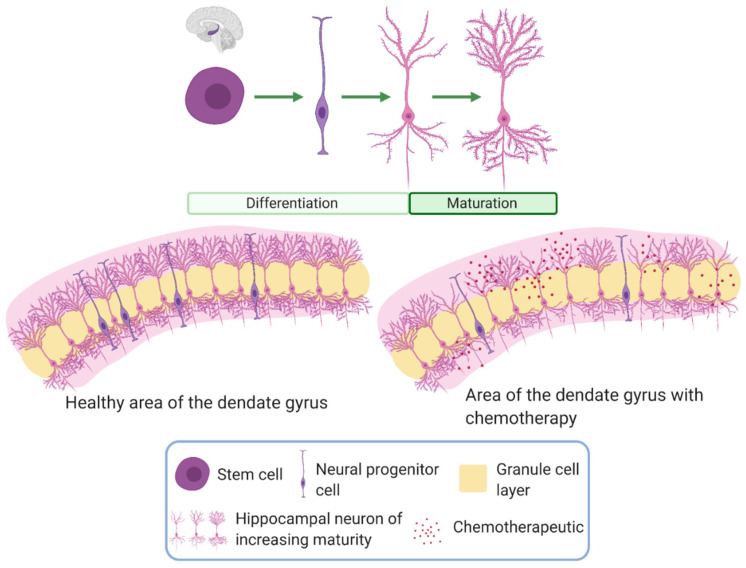
Neurogenesis and neural maturation with chemotherapy. The top image shows the progression of neurons from the stem cell to neural progenitor cell into neurons, which then mature, designated by the increase in processes and spines. The image below shows differences in neurogenesis observed in the dentate gyrus with chemotherapy. Chemotherapy results in a decrease in proliferative processes including neurogenesis and progenitor proliferations. This effect is seen through a reduction in neural progenitor cells and impaired maturation of neurons. Neurogenesis and neural maturation are reduced in *APOE4* individuals compared to those carrying the *APOE3* genotype. Stem cells from *APOE4* individuals produce fewer neural progenitor cells compared to *APOE3* individuals. Impaired neuron differentiation leads to slower maturation of neurons in *APOE4* individuals, resulting in less complex neurons.

In addition to neurogenesis, synaptogenesis is also impaired in the presence of *APOE4* in vivo and in primary cell cultures [165]. These findings support the well-established connection between *APOE4* and progressive cognitive decline, which is often marked by abnormalities such as loss of hippocampal synapses [166,167]. The *APOE*-dependent regulation of neurogenesis and synaptogenesis provides a rationale for investigating a possible association between different isoforms and susceptibility to chemotherapy-induced impairment of neurogenesis. Figure 2 provides a summary of how the *APOE* genotype could exacerbate the detrimental effects of chemotherapy on neurogenesis.

## 3. Interventions to Reduce the Severity of CRCI

The neurophysiological processes that are mediated by *APOE* would affect the risk and severity of CRCI. Table 2 reviews ways that mitigating the impact of *APOE4*, either reducing the detrimental influence of APOE4 or promoting the neuroprotective functions of APOE2/APOE3, may protect against CRCI. Some of these interventions are currently only being tested in vitro, while others are already being used as therapies for other diseases. APOE mimetic peptides reduce oxidative stress and inflammation by binding APOE receptors and are safe in humans in Phase I trials for spontaneous intracranial hemorrhage [168]. Agonists of the ABCA1 transporter increase APOE lipidation, which stimulates APOE function and reduces HDL inflammatory indices in high risk cardiovascular patients [169]. Several APOE-related therapeutic approaches are aimed at its effects on neuroinflammation, including cyclosporin A, non-steroidal anti-inflammatory drugs (NSAIDS) [139], and exercise [170]. Other approaches address APOE levels or APOE4 tertiary structure [171,172]. The connections between APOE biology and CRCI will aid in the development of therapeutic approaches against CRCI for the large fraction of the population who have inherited *APOE4*. In particular, preventative approaches before chemotherapy would be particularly promising. Furthermore, preventions that proved successful against the induced condition of CRCI may also be useful in other conditions, such as AD, which have a more prolonged onset.

## 4. Conclusions

There are a number of mechanisms that may play a role in the promotion of CRCI, and it is likely that a combination of these is responsible for observed debilitating effects. Each process likely varies in its impact depending on the cancer and the type of treatment. The *APOE4* genotype strongly influences several aspects of brain health, and these processes provide mechanistic insight into the causes of CRCI. *APOE* plays important roles in the function and integrity of the blood brain barrier. These effects would profoundly modify the extent of the neurotoxicity induced by oxidative stress and inflammation resulting from chemotherapeutic drugs. APOE also has more direct effects on neuroinflammation based on its anti-inflammatory nature and is involved in the fidelity of neurogenesis, which is also detrimentally affected by chemotherapy. Recognizing its importance in the response to cancer treatments emphasizes that identifying whether a patient is an *APOE4* carrier should be required before implementing a personalized regimen, as this could influence the susceptibility to, and severity of, possible neurological side effects. Moreover, there are a number of therapies that could alleviate some of the deleterious effects of chemotherapy that should be evaluated for co-administration to patients based on the impact it has on APOE function. Together, the ubiquitous roles of this essential component of many aspects of CNS health necessitates continued focus on the relationship between *APOE* genotype and CRCI.

## Figures and Tables

**Table 1:** Clinical and pre-clinical studies showing effects of *APOE4* on CRCI.

Study	Study Design	Results
**CLINICAL**		
Ahles et al., 2003 [23]. Long term survivors of breast cancer (8.8 years +/− 4.3 years post treatment) or lymphoma who had been treated with chemotherapy were given tests of cognition and stratified by the presence of at least one *APOE4* allele.	Breast cancer survivors (*n* = 51, age 55.9 +/− 8.8 years old), lymphoma survivors (*n* = 29 age 55.8 +/− 11.6). Together, 21% (*n* = 17) were *APOE4* carriers. A neuropsychological battery of tests assessed cognition, including verbal and spatial ability, verbal and visual memory, psychomotor function, and attention.	*APOE4* carriers scored significantly lower in visual memory and spatial ability, with a trend toward lower psychomotor function.
Ahles et al., 2014 [24]. Breast cancer patients treated with chemotherapy were assessed with cognitive tests prior to treatment at 1, 6 and 18 months post-chemotherapy, and were compared to breast cancer patients not treated with chemotherapy as well as healthy controls. Smoking history was also evaluated.	Chemotherapy treated breast cancer patients (*n* = 55, age 51.9 +/- 7.1; *APOE* carriers *n* = 14, 25%, had smoked *n* = 31, 56%). Non-chemotherapy treated breast cancer patients (*n* = 68, age 56.8+/− 8.3; *APOE4 n* = 18, 26%; smoked *n* = 43, 63%). Healthy controls (*n* = 43, age 53.0+/10.1; *APOE4 n* = 7, 16%; smoked *n* = 26, 60%). Individuals were evaluated for verbal ability and memory, visual and working memory, and processing speed.	Breast cancer patients who were *APOE4* carriers without a smoking history had significantly lower performance on processing speed and working memory compared to smokers and healthy controls. *APOE4* carriers without a smoking history who received chemotherapy had lower processing speed compared to those with a smoking history.
Mandleblatt et al., 2018 [5]. Newly diagnosed non-metastatic breast cancer survivors aged 60 years and older were matched controls without cancer. Cognitive measurements were collected before treatment at 12 and 24 months after treatment.	Breast cancer survivors: Chemotherapy +/− hormone therapy *n* = 80; *APOE4 n* = 12 (15%). Hormonal Therapy alone *n* = 201; *APOE4 n* = 41 (20%). Healthy controls *n* = 322; *APOE4* carriers *n* = 81 (25%). Individuals were tested for learning and memory (LM) and attention, processing speed and executive function (APE).	Hormonal Therapy alone: *APOE3* patients had no change in LM; *APOE4* patients had a short term decrease. There was no change in APE with *APOE* genotype. Chemotherapy: There was no change in APE in *APOE3* patients, but significant reduction in *APOE4* carriers.
Amidi et al., 2017 [25]. Testicular cancer patients were assessed for cognition and grey matter (GM) morphology after surgery but prior to further treatment, and 6 months later.	65 patients total, 22 received chemotherapy (age 31.9 +/− 9.4 years) and 43 did not (age 39.6 +/− 10.7). 20 of 61 (33%) patients with known *APOE* genotype were *APOE4* positive. There were 25 healthy controls (age 32.8 +/− 11.1) tested for attention and working memory, processing speed, auditory learning and memory, verbal fluency and executive functions.	Testicular cancer patients had greater cognitive decline than healthy controls (*p <* 0.05). *APOE4* carriers in cancer patients had significantly worse performance and had lower global composite score (*p <* 0.03) but did not have significant GM density changes.
**PRECLINICAL**		
Speidell et al., 2018 [41]. Female homozygous *APOE3* and *APOE4* mice at 4–6 months were treated with doxorubicin or saline, and subjected to cognitive tests involving mazes that measure spatial learning and memory, as well as MRI scans to measure structural brain changes.	*APOE3* (*n* = 18) and *APOE4* (*n* = 21) knock-in female C57BL/6J mice were used, most commonly used in studies of cognition based on good learning skills. Mice were treated with doxorubicin or saline, and spatial learning tests (Barnes maze) were performed 6 weeks post exposure, at 21 to 25 weeks of age.	*APOE4* mice treated with doxorubicin had significantly reduced spatial and learning memory compared to *APOE3* mice, which showed no impairment. There were significant MRI changes in the cortex and hippocampus after treatment, with similar patterns in both *APOE* genotypes, more pronounced in *APOE4.*
Demby et al., 2020 [42]. Female aged (12 months old) homozygous *APOE3* or *APOE4* mice were treated with doxorubicin or saline, and then subjected to cognitive and behavioral assays, and MRI scans were performed to detect regional brain volume differences.	*APOE3* (*n* = 30) and *APOE4* (*n* = 31) knock-in female C57BL/6J mice were used, and measures taken at 31–35 weeks post-exposure. Spatial and memory tests were analyzed via the Barnes maze, and tissue sections stained for markers of AD pathogenesis.	*APOE3* mice were unaffected but *APOE4* mice had significant impairment in spatial learning after doxorubicin treatment. Doxorubicin impaired spatial memory in both genotypes. There were no changes in AD marker expression.

**Table 2:** Potential interventions to reduce the severity of cancer related cognitive impairments.

Treatment	Mechanism	Experimental Stage	Reference
APOE mimetics	Bind APOE receptors	Pre-clinical: alleviated CNS damage induced by ischemic stroke in wildtype mice	[173]
		Pre-clinical: reduced inflammation and oxidative stress, improved cognition in wildtype mice	[174]
		Phase I human trials: completed as treatment for spontaneous intracranial hemorrhage	[168]
		Pre-clinical: inhibited BBB impairment following subarachnoid hemorrhage (SAH), reduced inflammation and improved cognition in wildtype mice	[175]
ABCA1 agonists	Increase ABCA1 activity and hence APOE4 lipidation. Increase APOE levels	Pre-clinical: alleviated synaptic impairment and improved cognition in APOE4 mice	[176]
		In vitro: mitigated inhibition of APOE secretion by AD pathogenic protein Aβ in vitro	[177]
		Clinical Trials: showed HDL inflammatory index reduced in high risk cardiovascular patients	[169,178]
Retanoic X receptor agonists	Increase APOE levels and lipidation to increase activity	Pre-clinical: effective in reducing cognitive decline in wildtype mice	[179,180,181]
		Clinical: Approved for use in humans as a cancer treatment	[182]
APOE4 structure correctors	Alleviate pathological intramolecular domain interactions	In vitro: increased neurite outgrowth in neurons and improved mitochondrial activity in APOE4 cells to resemble APOE3 cells	[183,184,185]
Cyclosporin A	Inhibits pro-inflammatory cyclophilin A to reduce BBB breakdown	Pre-clinical: reduced BBB leakage in APOE4 mice	[57]
		Clinical: used to prevent organ rejection in humans	[186]
Exercise	Reduce rate of cognitive decline by unknown mechanism	Post hoc analysis: more beneficial for APOE4 carriers	[187]
APOE peptide antagonist	Disrupts interaction between APOE and Aβ and reduces inflammation and AD pathology	Pre-clinical: reduces inflammation and AD pathology induced by APOE-Aβ binding in AD mouse model	[188,189]
Antisense oligonucleotides	Reduce APOE levels	Pre-clinical: reduced AD pathology in an AD mouse model	[172]
Anti-inflammatory-NSAIDs	Reduces inflammation, increases neuronal complexity	Pre-clinical: causes APOE4 mice to more closely resemble APOE3 mice in AD model	[139]
CRISPR/Cas9	Editing APOE4 to either APOE2 or APOE3, or editing APOE3 to the protective Christchurch mutation	In vitro: corrects APOE4-dependent dysfunction in neurons, astrocytes, and microglia	[130,190,191]
